# 413. Living with Long COVID: A Study on Persistent Symptoms and Vaccination Outcomes Two Years After COVID-19 Infection

**DOI:** 10.1093/ofid/ofad500.483

**Published:** 2023-11-27

**Authors:** Yoonjung Kim, Shin-Woo Kim, Sohyun Bae, Hyun-Ha Chang

**Affiliations:** Kyungpook national university hospital, Daegu, Taegu-jikhalsi, Republic of Korea; School of Medicine, Kyungpook National University, Daegu, Taegu-jikhalsi, Republic of Korea; Division of Infectious Diseases, Department of Internal Medicine, Kyungpook National University Hospital, School of Medicine, Kyungpook National University, Daegu, Republic of Korea, Taegu, Taegu-jikhalsi, Republic of Korea; Division of Infectious Diseases, Department of Internal Medicine, School of Medicine, Kyungpook National University, Daegu, Korea, Daegu, Taegu-jikhalsi, Republic of Korea

## Abstract

**Background:**

Understanding the characteristics of long COVID and the effect of COVID-19 vaccinations on patients after 24 months of COVID-19 infection is crucial for effective disease management. This study aims to determine the prevalence of long COVID and its impact on lifestyle 24 months after COVID-19 infection.

**Methods:**

This prospective cohort study included 235 adult patients diagnosed with COVID-19 between February 17, 2020, and March 24, 2020. The participants visited the study hospital four times, at 6, 12, 18, and 24 months after acute COVID-19 infection, to assess their symptoms, quality of life, and mental health. The clinical characteristics, self-reported long COVID symptoms, EuroQol 5-dimension 5-level (EQ-5D-5L) index, Korean versions of the Patient Health Questionnaire-9 (PHQ-9), Posttraumatic Stress Disorder Checklist-5 (PCL-5-K), and Generalized Anxiety Disorder-7 (GAD-7) scale scores were investigated 24 months after acute COVID-19 infection. The presence or absence of 24-month long COVID symptoms and COVID-19 vaccination, initial disease severity, and clinical characteristics over time were compared and analyzed.

**Results:**

Out of the 235 patients, 121 (51.5%) completed the study visits, and 75 (62.0%) still experienced long COVID symptoms 24 months after acute infection. Fatigue, amnesia, difficulty concentrating, and insomnia were the most common symptoms (Figure 1). The frequency of neuropsychiatric symptoms did not differ based on vaccination status or the number of doses received. However, patients with long COVID symptoms had significantly higher scores on measures of anxiety and depression than those without long COVID symptoms. The quality of life for the participants improved over time but still affected 32.2% of respondents, particularly in the anxiety/depression dimension.

**Figure:**
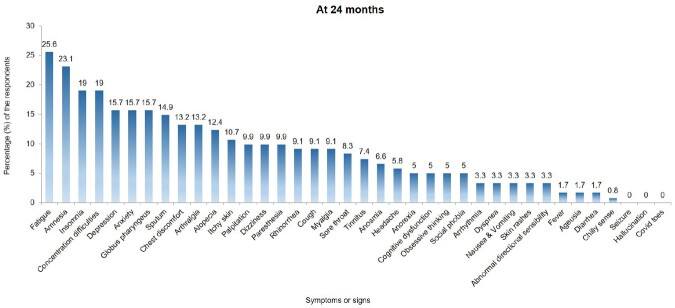

**Conclusion:**

This study highlights that long COVID symptoms can persist up to 24 months after acute COVID-19 infection, affecting mental health and quality of life. Although COVID-19 vaccination did not seem to impact the frequency of neuropsychiatric symptoms, further research is needed to fully understand the long-term impact of COVID-19 and the efficacy of COVID-19 vaccinations on long COVID symptoms.

**Disclosures:**

**All Authors**: No reported disclosures

